# Risk of Mortality Among Adult Females Diagnosed with Traumatic Brain Injury in an Academic Medical System

**DOI:** 10.1177/08977151251361700

**Published:** 2025-08-01

**Authors:** Bernadette A. D’Alonzo, Abigail C. Bretzin, Rebecca B. Morse, Silvia P. Canelón, Douglas J. Wiebe, Andrea L.C. Schneider, Mary Regina Boland

**Affiliations:** ^1^ Department of Neurology, Perelman School of Medicine, University of Pennsylvania, Philadelphia, Pennsylvania, USA.; ^2^ Department of Biostatistics, Epidemiology and Informatics, Perelman School of Medicine, University of Pennsylvania, Philadelphia, Pennsylvania, USA.; ^3^ Injury Prevention Center, Department of Emergency Medicine, University of Michigan, Ann Arbor, Michigan, USA.; ^4^ Department of Emergency Medicine, Perelman School of Medicine, University of Pennsylvania, Philadelphia, Pennsylvania, USA.; ^5^ Department of Epidemiology, University of Michigan, Ann Arbor, Michigan, USA.; ^6^ Institute for Biomedical Informatics, University of Pennsylvania, Philadelphia, Pennsylvania, USA.; ^7^ Center for Excellence in Environmental Toxicology, University of Pennsylvania, Philadelphia, Pennsylvania, USA.; ^8^ Department of Biomedical and Health Informatics, Children’s Hospital of Philadelphia, Philadelphia, Pennsylvania, USA.; ^9^ Data Science, Department of Mathematics, Boyer School of Mathematics, Natural Sciences & Computing, Saint Vincent College, Latrobe, Pennsylvania, USA.

**Keywords:** females, lifespan, mortality risk, severity, traumatic brain injury

## Abstract

The objective of this retrospective cohort study was to evaluate mortality risk over five years among 6,432 female patients with a health care encounter diagnosis of TBI from hospitals and outpatient clinics within a university health system. We used TBI severity, defined by the Centers for Disease Control and Department of Defense/Veterans Affairs: mild, moderate/severe/penetrating, indeterminate severity. To determine patient death, we used death in a Penn Medicine facility and linkage to the Social Security Death Index. We used Cox proportional hazards models adjusted for age at the time of TBI diagnosis, race, and encounter type to estimate associations of TBI severity with mortality risk. We evaluated interactions with encounter type and age, and stratified results by inpatient/outpatient and age group (≥65 years). Median age was 47 years (25th–75th percentiles: 29–63). Patients were most commonly self-reported White race (*n* = 4,126, 64.0%), and diagnosed at an outpatient encounter (*n* = 5,099, 79.3%; among them, 1–2% urgent/emergent). Median follow-up time was 4.22 years (IQR, 2.3–4.9 years). Overall, 2.9% (*n* = 185) of patients died within five years of injury. Compared with mild TBI, mortality risk over five years was 2.06 times higher (95% CI = 1.27–3.33) for moderate/severe/penetrating TBI, and 1.54 times higher (95% CI = 0.98–2.42) for indeterminate TBI. Associations were attenuated among females with inpatient encounter type and those aged 65 years or older. Our results demonstrate that TBI severity affects survival among females, and this differs by encounter type and age. Findings motivate future, more focused research into the dynamics of TBI among females.

## Introduction

### Background

Traumatic brain injury (TBI) is an acute injury that often results in life-altering consequences for the injured individual and their families.^1,2^ TBI is common in the United States (US), with an estimated 1.7 million Americans experiencing a TBI annually.^3^ Described as a “silent epidemic,”^4^ TBI is a pervasive and significant public health concern, and yet, our understanding of its epidemiology is limited, particularly among females within large academic medical system (hospital and outpatient) settings^5^ who represent an understudied group across the TBI literature.^6,7^

The known leading causes of TBI include motor vehicle and motorcycle crashes, falls, participation in contact sports (most notably, football and soccer), and military or other athletic training, which are linked to mechanisms thought to typically, predominantly affect males.^8^ Over time, TBI surveillance and research in the United States have focused on males from younger, athletic, and combat-veteran populations.^9^ As a result, much of the TBI research literature, which relies on these data and, in turn, informs the clinical management of TBI patients is based on male samples and focused on younger age groups.^7,10–12^

The ability to elucidate differences in severity and survival among females with TBI is a limitation of many existing clinical and neurological studies that rely on small samples.^7,13–15^ In the early 2020s, initiatives to study the risk, burden, and clinical trajectories of female athletes and combat-veterans with TBI have emerged.^16,17^ However, by focusing predominantly on these specialized populations, researchers may be excluding additional characteristics of the patient and/or TBI, such as mechanisms of injury that occur among non-athletes and non-veterans. Because of these gaps, the nature of TBI and associated outcomes among females in hospital and outpatient settings are not well understood.^18^

Several recent studies have reported on the impact of acute TBI on long-term survival and found higher mortality rates over time among TBI patients from hospital cohort settings compared with non-TBI controls and the U.S. general population.^19–21^ Important covariates identified in previous work include age,^19,20^ sex,^19^ and mechanism of injury.^8,22^ In addition, one study classified and stratified their analysis according to TBI diagnosis certainty using the Mayo classification for TBI diagnosis^19^ (i.e., definite, probable, and possible TBI), and found that with greater certainty of TBI diagnosis, mortality risk increased.^19^ Still, studies with male and female mixed-cohorts largely adjust for patient sex rather than present results explicitly among female patients. Additional research is needed to determine the survival rates among females with varying TBI severity and to evaluate for differences by age.

Here, we explore the risk of mortality among females who have been diagnosed with a TBI using electronic health record (EHR) data, which remain an underutilized resource for better understanding TBI in hospital and outpatient settings. Overall, TBI awareness, resources, and research progress in females have lagged and contributed to gaps in knowledge regarding TBI survival. The purpose of this study is to address this gap and estimate the risk of mortality over five years following a TBI diagnosis among a cohort of females from a large university health system.

## Materials and Methods

### Data source

Our study source population consisted of an initial set of 1,060,100 female patients with visits at inpatient or outpatient clinic settings at Penn Medicine between 2010 and 2017. These data were obtained from the EHR within four different hospitals of the Penn Medicine system: the Hospital of the University of Pennsylvania, the Pennsylvania Hospital, Penn Presbyterian Hospital (Level 1 trauma center), and Chester County Hospital, along with several ancillary outpatient clinics and centers.

The Institutional Review Board (IRB) at Penn Medicine approved this study (IRB protocol #851731 and #828000), which is a retrospective analysis of existing clinical records. This study is reported per the Strengthening the Reporting of Observational Studies in Epidemiology (STROBE) guidelines.

### TBI definitions

We identified patients as having a diagnosis of TBI if they met our definition of TBI,^23^ which incorporated both the Centers for Disease Control (CDC)^24^ and U.S. Department of Defense (DOD)^25^/Department of Veterans Affairs (VA)^26–28^ surveillance case definitions to identify patients with TBI using a combination of International Classification of Diseases, 9th Revision, Clinical Modification (ICD-9-CM) and ICD, 10th Revision (ICD-10) billing codes. Our method has been described and evaluated previously.^23^ A full list of all ICD9 and ICD10 codes used to define TBI and TBI severity from the EHR in our cohort has been published previously^23^ and is available on GitHub (https://github.com/bolandlab/TBI_Definition) to enhance both the reproducibility and accessibility of our work. This definition allowed us to apply the DOD/VA TBI severity classifications (mild, moderate/severe/penetrating, indeterminate severity) to assign TBI severity.^26–28^

### Mortality

We used two sources of information to determine patient death: death in one of our Penn Medicine facilities and the Social Security Death Index (SSDI).^29^ Penn Medicine periodically links the SSDI with their patient records to determine if patients are currently living or dead for insurance purposes. Therefore, we have death information for all patients with valid social security numbers that were treated at Penn Medicine. An issue with the SSDI is the potential for lag between the death of the patient and that information being provided to the SSDI. For this reason, we censored patients at a fixed date, which was a minimum of six months after the latest TBI diagnosis date in our cohort.

### Cohort sample selection

**Appendix Fig. A1.**
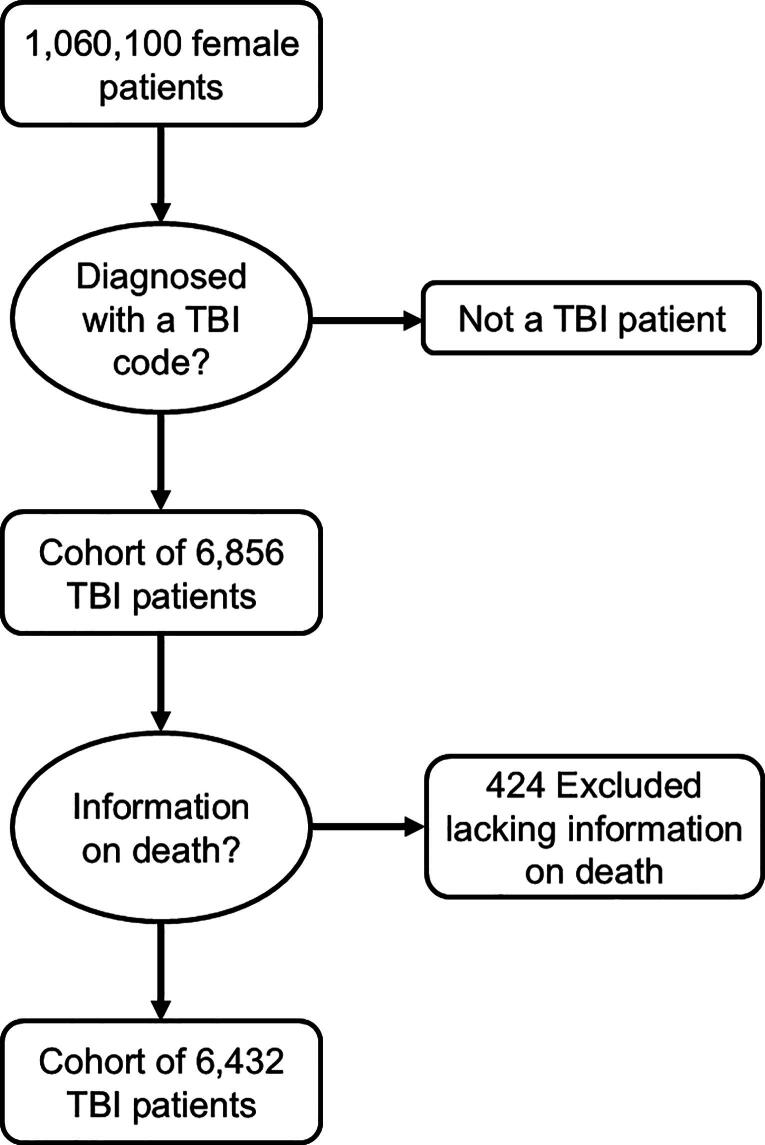
Study cohort sample selection flowchart; female patients with traumatic brain injury (TBI) from Penn Medicine.

From our initial source population of female patients treated within Penn Medicine, we determined our cohort of TBI patients. We first identified patients who had a health care encounter with an associated TBI-related ICD code. We excluded patients with only Glasgow Coma Scale (GCS) ICD code diagnoses if they did not have a corresponding injury code, as these may be associated with other medical diagnoses and not TBI. This yielded a preliminary cohort of *n* = 6,856 TBI patients. We then excluded 424 TBI patients where information on death (both deceased date and alive/deceased status) was missing. This yielded a final cohort of *n* = 6,432 TBI patients for analysis. Appendix Fig. A1 depicts an overview of our cohort sample selection process.

### Covariates

We obtained covariates used in our analytic models from the Penn EHR, including age, race, Hispanic ethnicity, injury mechanism, encounter type (inpatient/outpatient), and provider department where the TBI diagnosis was made. Age was defined at the time of the TBI diagnosis encounter. We also verified age by determining the time between the date of birth and the TBI diagnosis encounter to ensure this value matched the patient’s age recorded at the time of the TBI diagnosis encounter.

### Statistical analysis

We present descriptive demographic and clinical characteristics as median and 25th–75th percentiles for continuous variables and number and percent for categorical variables. We conducted survival analysis using the time of TBI health care encounter to outcome death (in years) by TBI severity category (mild, moderate/severe/penetrating, indeterminate severity), with administrative censoring at 5 years following TBI diagnosis. In our study, we set the first date of TBI as the first diagnosis date of the most severe TBI diagnosis. We used Kaplan–Meier curves and log-rank tests to examine overall differences in survival time by TBI severity.^23^ We used Cox proportional hazards regressions to estimate hazard ratios for mortality risk. Our main exposure of interest was TBI severity. We examined complementary log–log plots for violations of the proportional hazards assumption and assessed the correlation between scaled Schoenfeld residuals and time using the Schoenfeld test.^23^ We constructed models between TBI severity and outcome time to death, adjusting for age at time of TBI diagnosis, race, and encounter type as covariates of interest. We conducted two sensitivity analyses. First, we tested for interaction between TBI severity and encounter type (inpatient/outpatient) and stratified our analysis by encounter type. Second, we tested for interaction between TBI severity and age, and stratified by age group (≥65 years). We constructed our models in R version 4.0.3 using the Survival and survminer packages,^30^ and created Kaplan–Meier curves with ggsurvplot.^31^

## Results

We identified 6,432 female patients with a medical encounter diagnostic code for TBI using the combined CDC and DOD/VA definitions for TBI^24–28^ between January 1, 2010, and August 15, 2017, at Penn Medicine (Table 1).^23^ TBI severity was most often of indeterminate severity (*n* = 2,766, 43.0%) followed by mild severity (*n* = 2,629, 40.9 %). Median follow-up time was 4.22 years (IQR, 2.3–4.9 years). Median age at time of TBI diagnosis encounter was 47 years (25th–75th percentiles: 29–63) and patients were most commonly of self-reported White race (*n* = 4,126, 64.0%). Regarding TBI mechanisms of injury, 13% of TBIs (*n* = 819) resulted from a fall and 3.1% (*n* = 184) resulted from a collision/crash, however, the etiologies of most (*n* = 5,019, 78.0%) could not be determined using ICD codes. The encounter type for the majority of patients was outpatient (*n* = 5,099, 79.3%), with roughly 1–2% classified as urgent or emergent. In total, 2.9% (*n* = 185) died within five years of injury.

**Table 1.** Demographic and Clinical Characteristics of Patients in Study

	No. (%)			
	All participants	Mild	Moderate/severe/penetrating	Indeterminate
No. of patients	6,432	2,629	1,037	2,766
Demographics				
Age, median (25th–75th percentiles)^a^, years	47 (29–63)	40 (24–56)	58 (40–72)	49 (32–64)
Age group, ≥65 years	1,443 (22.4)	388 (14.8)	402 (38.8)	653 (23.6)
Race				
Asian	186 (2.9)	65 (2.5)	36 (3.5)	85 (3.1)
Black/African American	1,668 (26.0)	601 (23.0)	296 (28.5)	771 (28.0)
White	4,126 (64.0)	1,768 (67.0)	633 (61.0)	1,725 (62.0)
Other	217 (3.4)	94 (3.6)	33 (3.2)	90 (3.3)
Unknown	235 (3.7)	101 (3.8)	39 (3.8)	95 (3.4)
Hispanic ethnicity	182 (2.8)	61 (2.3)	27 (2.6)	94 (3.4)
Clinical characteristics				
Injury mechanism				
Collision/crash	185 (2.9)	71 (2.7)	76 (7.3)	38 (1.4)
Fall	819 (13.0)	187 (7.1)	313 (30.2)	319 (12.0)
Physical assault	70 (1.1)	23 (0.9)	14 (1.4)	33 (1.2)
Other	339 (5.3)	173 (6.6)	55 (5.3)	111 (4.0)
Unknown	5,019 (78.0)	2175 (82.7)	579 (55.8)	2,265 (81.9)
Encounter type^b^				
Inpatient	1,333 (20.7)	256 (9.7)	516 (49.8)	561 (20.3)
Outpatient	5,099 (79.3)	2,373 (90.3)	521 (50.2)	2,205 (79.7)
*Outpatient, emergency/urgent*	83 (1.6)	44 (1.7)	7 (1.3)	32 (1.5)
Provider department				
Neurology/neurosurgery	1,257 (19.5)	418 (15.9)	427 (41.2)	413 (14.9)
Emergency medicine/trauma service	327 (5.2)	138 (5.2)	113 (10.9)	76 (2.7)
General practitioner	3,142 (48.8)	1608 (61.2)	200 (19.3)	1259 (52.1)
Specialist	1,706 (26.5)	465 (17.7)	297 (28.6)	291 (30.8)
Deceased				
Yes	175 (2.7)	26 (1.0)	81 (7.8)	68 (2.5)

^a^ Age at traumatic brain injury (TBI).

^b^ <1% were both inpatient and outpatient; included as inpatient.

**FIG. 1.**
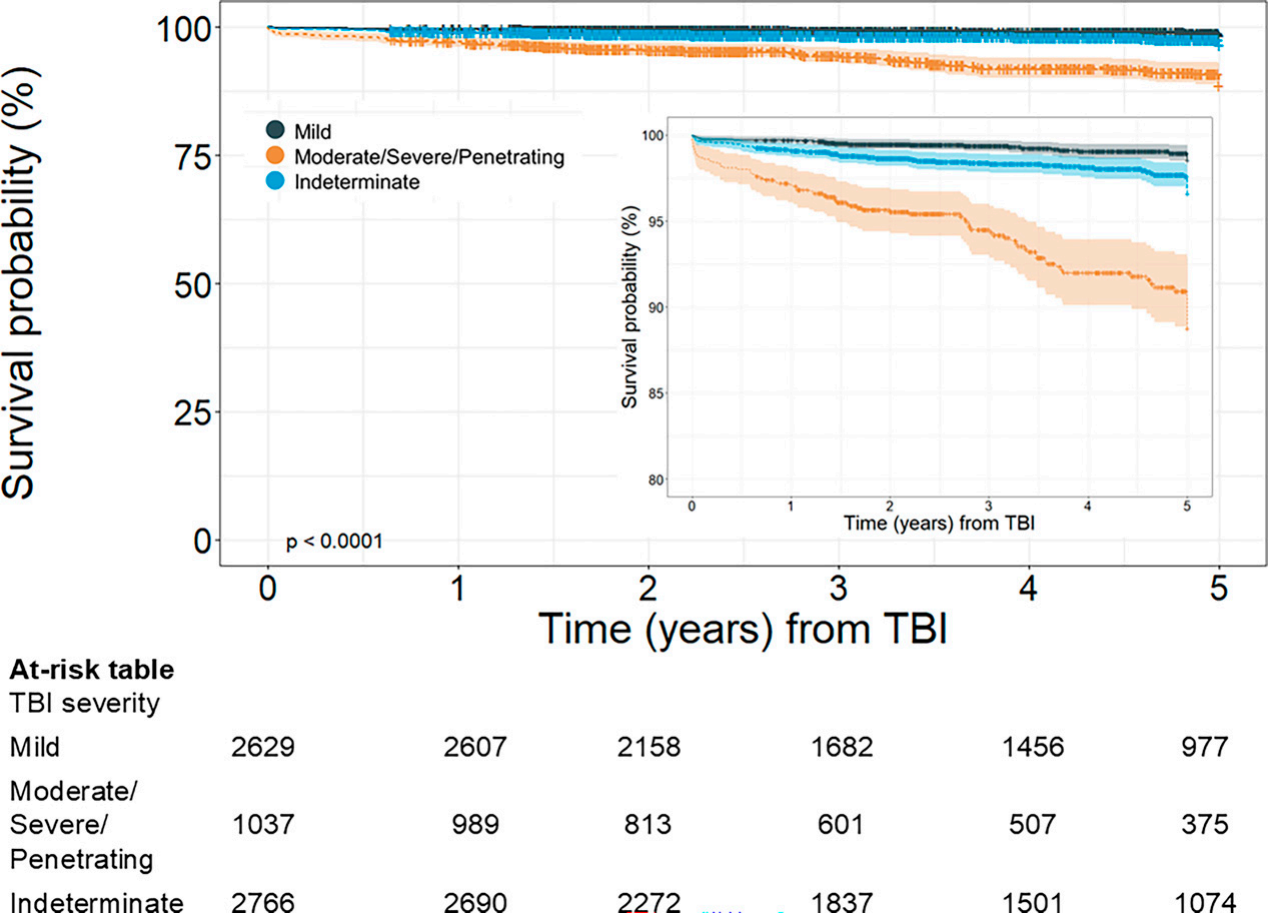
Kaplan–Meier curves depicting time from traumatic brain injury (TBI) diagnosis encounter to death by TBI severity. The shaded bands around each line represent the 95% confidence intervals (CIs).

**FIG. 2.**
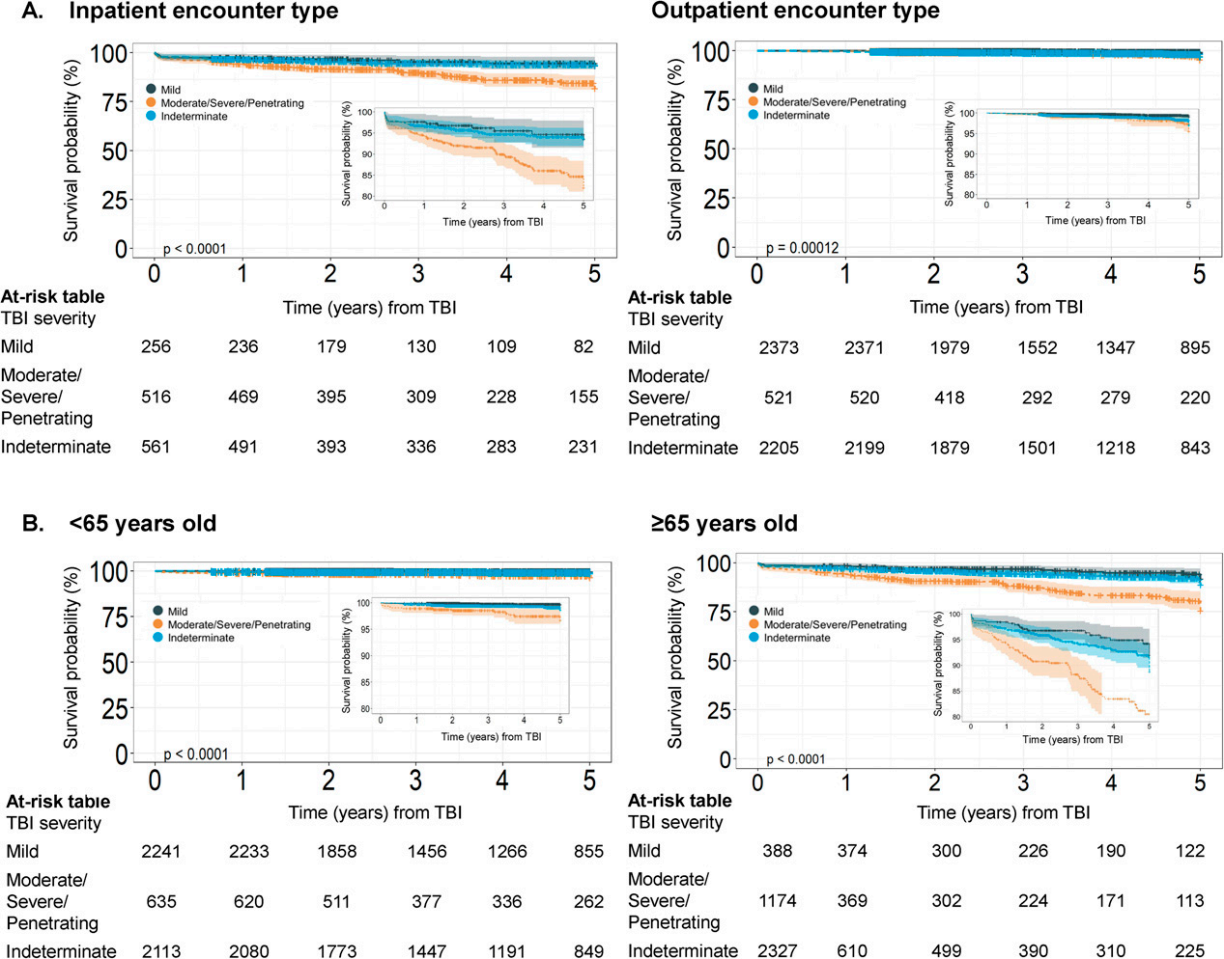
Kaplan–Meier curves depicting time from traumatic brain injury (TBI) diagnosis encounter to death by TBI severity among patients (**A**) with inpatient compared with outpatient encounter type, and (**B**) <65 years old compared with ≥65 years old. The shaded bands around each line represent the 95% confidence intervals (CIs).

Cumulative survival was consistently lower for moderate/severe/penetrating compared with mild severity (log-rank, *p* < 0.0001; Fig. 1). In adjusted models, compared with mild TBI, mortality risk was 2.06 times higher (95% CI = 1.27–3.33) for moderate/severe/penetrating TBI, and 1.54 times higher (95% CI = 0.98–2.42) for Indeterminate TBI severity (Table 2). Formal testing for multiplicative interaction between TBI severity and encounter type (inpatient/outpatient) was not statistically significant (*p* = 0.57). Among those with inpatient encounter type-based diagnoses, compared with mild TBI, mortality risk was 1.72 times higher (95% CI = 0.92–3.21) for moderate/severe/penetrating TBI, and 1.14 times higher (95% CI = 0.58–2.44) for indeterminate TBI severity, adjusting for age and race. Among outpatient type visit-based diagnoses, compared with mild TBI, mortality risk was 2.31 times higher (95% CI = 1.08–4.94) for moderate/severe/penetrating TBI, and 1.60 times higher (95% CI = 0.86–2.97) for indeterminate TBI severity, adjusting for age and race (Table 3, Fig. 2).

**Table 2.** Cox Proportional Hazards Regression-Breslow Methods for Ties, Hazard Ratios for Association Between TBI and Risk of Mortality Within Five Years (*N* = 6,432)

	HR	95% CI
TBI severity level		
Mild	Ref	Ref
Moderate/severe/penetrating	2.06	1.27–3.33
Indeterminate severity	1.54	0.98–2.42
Age	1.06	1.05–1.07
Race		
White	Ref	Ref
Asian	0.42	0.10–1.71
Black/African American	0.79	0.56–1.13
Other	1.15	0.47–2.83
Unknown	1.44	0.77–2.68
Encounter type		
Outpatient	Ref	Ref
Inpatient	3.05	2.14–4.35

HR, hazard ratio; TBI, traumatic brain injury.

**Table 3.** Cox Proportional Hazards Regression-Breslow Methods for Ties, Hazard Ratios for Association Between TBI and Risk of Mortality Within Five Years (*N* = 6,432)

	HR	95% CI	*p* value for interaction
Stratified by encounter type^a^			*p* = 0.57
Inpatient			
Mild	Ref	Ref	
Moderate/severe/penetrating	1.72	0.92–3.21	
Indeterminate severity	1.14	0.58–2.24	
Outpatient			
Mild	Ref	Ref	
Moderate/severe/penetrating	4.19	1.97–8.92	
Indeterminate severity	2.90	1.57–5.36	
Stratified by age group, 65 and older^b^			*p* = 0.006
<65 years			
Mild	Ref	Ref	
Moderate/severe/penetrating	4.35	1.60–11.79	
Indeterminate severity	2.33	0.91–5.94	
≥65 years			
Mild	Ref	Ref	
Moderate/severe/penetrating	1.99	1.16–3.41	
Indeterminate severity	1.37	0.81–2.31	

^a^ Model adjusted for age, race.

^b^ Model adjusted for encounter type, race.

HR, hazard ratio; TBI, traumatic brain injury.

Formal testing for multiplicative interaction between TBI severity and age was statistically significant (*p* < 0.01). Among those age 65 and older, compared with mild TBI, mortality risk was 1.99 times higher (95% CI = 1.16–3.41) for moderate/severe/penetrating TBI and 1.37 times higher for (95% CI = 0.81–2.31) for indeterminate TBI severity, adjusting for race and encounter type. Among those younger than 65 years, compared with mild TBI, mortality risk was 4.35 times higher (95% CI = 1.60–11.79) for moderate/severe/penetrating TBI and 2.33 times higher for (95% CI = 0.91–5.94) for indeterminate TBI severity, adjusting for race and encounter type (Table 3, Fig. 2).

## Discussion

The purpose of this study was to investigate TBI severity and the role of demographic and clinical features in contributing to the risk of death over five years post-injury among females. We found that compared with mild TBI, females with moderate/severe/penetrating TBI were significantly more likely to have died within five years of TBI diagnosis. Our findings extend the existing literature on mortality risk among females with TBI.^21,32,33^

This study represents a key contribution to the literature by focusing on an understudied cohort of females with TBI. We leverage data from the Penn Medicine health system, which serves a predominantly urban and suburban community-patient population, and includes a level 1 trauma center and quaternary referral center.^5^ Our cohort includes female patients from both inpatient and outpatient (largely outpatient, 79.3%) visits with the most common mild and indeterminate TBI severity. We found a dose-response pattern in the risk of death within five years by TBI severity, whereby the likelihood of death was nearly threefold higher for those with moderate/severe/penetrating TBI compared with mild TBI. This finding confirms and extends existing research, which has demonstrated increased all-cause mortality risk among individuals with moderate/severe TBI compared with those with mild TBI or without TBI.^34–38^

Interestingly, we found that patients with TBI that was unable to be classified into a severity type using the established DOD/VA algorithm (i.e., indeterminate severity) had a higher risk of mortality (HR = 1.54, 95% CI = 0.98–2.42), although this effect was not statistically significant. Still, this may suggest that these TBIs were more severe than mild TBIs. Besides TBI severity, further work is needed to understand the underlying mechanisms and role of potential comorbidities (and confounders) on associations of TBI with mortality, which reflect the clinical complexity of patients as having a TBI with complications or without, so that interventions and treatments may be developed. In addition, this finding may indicate a clinical practice issue, whereby patients are coded with indeterminate severity TBIs due to other factors that are also contributing to their elevated risk of mortality within five years.

Our findings build upon several prior studies that demonstrate decreased survival associated with TBI among patients using inpatient and outpatient health records data.^38^ Our results also align with recent findings from the Atherosclerosis Risk in Communities (ARIC) Study, an ongoing community-based cohort study of participants aged 45–64 from four states in the United States.^39^ Results from the ARIC cohort showed an approximate two-fold increase in mortality risk associated with head injury compared with those without; occurring a median of 4.4 years after head injury.^21^

The current study also includes female patients across a broad age spectrum (median age: 47 years, 25th–75th percentiles: 29–63). Our findings confirm results of some descriptive studies^8,22^ that demonstrate a bimodal distribution in the TBI incidence, where TBI incidence is highest during young adulthood and among older adults (aged 75+ years).^8,22^ We found a statistically significant interaction between TBI severity and age (*p* < 0.01), and in our analysis stratified by age group (≥65 years), we found compared with mild TBI, higher risk of mortality within five years among moderate/severe/penetrating and indeterminate severity TBI that was stronger among younger (<65 years) compared with older females. The reasons behind this finding are likely multifactorial and may reflect differences in baseline health status, injury mechanism, and severity.^21,37^ Further research focused on better understanding these relationships among females is needed to develop interventions targeted at improving outcomes among younger females with TBI.

TBI studies have focused primarily on male patients with often occupational-related TBI, resulting from participation in military and contact sports activities.^7–12^ Patients in these cohorts are frequently young, otherwise healthy individuals (with the exception of the veteran population, where there may be older patients). Therefore, results (and the case definitions and guidelines that they inform) have tended to be oriented toward males in these specialized populations. The current study focuses on a hospital and outpatient cohort of females to highlight this underrepresented population. Many previous studies have been limited to examining sex as a covariate or effect modifier of long-term survival.^7,21^ Here, the size of our all-female, adult cohort enabled us to evaluate survival among females experiencing TBIs of different severity types, and by encounter type and age.

Sex and gender differences have been explored and observed in a wide range of health outcomes, often due to (a) under-resourced research on females or individuals identifying as women, and also (b) disparities in care due to discrimination and bias.^40^ Specifically for TBI, emerging evidence shows sex and gender differences in terms of TBI incidence, diagnosis, and short- and long-term outcomes.^7,10,11,13,14^ The reasons behind this are likely multifactorial and may include differences in reporting behaviors, such as how TBI symptoms are described. However, they may also reflect clinical diagnostic biases, such as ascribing symptoms to other causes in females (e.g., menstrual disorders, menopause, migraines) to explain common symptoms of TBI (e.g., light-headedness, headache, mood changes).^33^ These subtle differences may manifest more broadly in reporting behaviors of the injury itself, symptoms, and lasting sequelae.^7,10,12,13,33^ In our research, we highlight females as a group under-represented in the TBI literature. Here, we use EHR data where demographic characteristics (including sex) are updated at each visit to capture individuals who may identify differently at different times.

### Limitations

There are several limitations of our work that will also spur future research directions. Our cohort is comprised of patients from hospitals, clinics, and centers within one large health system in Philadelphia, PA, and so our results may only be generalizable to patients in the greater Philadelphia area. However, Philadelphia includes one of the most diverse populations in the country with 57% of the population comprised of women, 40% individuals of self-reported Black race, and 16% of Hispanic or Latino ethnicity, and a wide range of socioeconomic statuses represented.^5^ We used a definition of TBI that combined two existing and established TBI case definitions from the CDC and DOD/VA, and some additional codes using both ICD-9 and ICD-10 versions.^23^ There are inherent limitations to using ICD codes as a proxy for TBI diagnosis or any disease diagnosis. Prior literature suggests that ICD code-based TBI definitions are susceptible to misclassification, particularly for mild TBI, with validation studies reporting 55–72% sensitivity and 80–85% specificity, depending on the ICD-code definition used.^41,42^ In this study, we used standard ICD codes as used by the CDC and DOD/VA to identify TBI patients; we did not re-evaluate these code sets but employed them on our cohort, as discussed in our previous work.^23^ In addition, we did not adjust for other medical comorbidities in this analysis and we recognize this as an important component of future work. While acknowledging these limitations, we lean on both the CDC and DOD/VA ICD definitions, which have been validated.^23,41,42^ Our study consists of a retrospective analysis of existing patient data obtained from EHR data and this introduces several limitations. The date of TBI diagnosis might not be the actual date of TBI injury. For most patients, the date of diagnosis will be close to the date of injury, but for some patients with prolonged sequelae, there might be a considerable treatment delay that is not captured in structured EHR data. Furthermore, the mechanism of injury was not present in roughly 70% of our sample. Still, our finding that falls was the most commonly recorded injury mechanism is reflective of the wide age range of patients in our sample, and is consistent with earlier literature and CDC surveillance statistics, as falls remain the leading cause of TBI in older adults.^8,24^ Further analysis imputing mechanism of injury from clinical notes using natural language processing methods is an important future work to determine opportunities for intervention.

## Conclusions

In this study, we evaluated the five-year all-cause survival among females diagnosed with at hospitals, emergency rooms, and outpatient clinics within a large university health system. We focus on females with TBI in this study, highlighting them as an underrepresented group, whereas many prior studies have focused on males exclusively. Specifically, we investigated the role of TBI severity and demographic and clinical features, such as encounter type and age, in contributing to the risk of death within five years. We found that compared with females with mild TBI, those with moderate/severe/penetrating TBI were significantly more likely to have died within five years of TBI diagnosis encounter. Our findings demonstrate that TBI severity affects survival among females, and this differs across the lifespan in females. Our study motivates future, more focused research into TBI among female populations.

## Transparency, Rigor, and Reproducibility Statement

Data in this study are not publicly available. We will make all relevant code and other shareable resources available on our GitHub page: https://github.com/bolandlab.

## Abbreviations Used

ARIC: Atherosclerosis Risk in Communities Study
CDC: Centers for Disease Control
DOD: Department of Defense
EHR: Electronic health record
GCS: Glasgow Coma Scale
ICD-9-CM: International Classification of Diseases, 9th Revision, Clinical Modification
ICD-10: ICD, 10th Revision
IRB: Institutional Review Board
IQR: Interquartile Range
STROBE: Strengthening the Reporting of Observational Studies in Epidemiology
SSDI: Social Security Death Index
TBI: Traumatic Brain Injury
US: United States
